# GaN Micromechanical Resonators with Meshed Metal Bottom Electrode

**DOI:** 10.3390/ma8031204

**Published:** 2015-03-17

**Authors:** Azadeh Ansari, Che-Yu Liu, Chien-Chung Lin, Hao-Chung Kuo, Pei-Cheng Ku, Mina Rais-Zadeh

**Affiliations:** 1 Department of Electrical Engineering and Computer Science, University of Michigan, Ann Arbor, MI 48109, USA; E-Mails: azadans@umich.edu (A.A.); peicheng@umich.edu (P.-C.K.); 2 Department of Photonic & Institute of Electro-Optical Engineering, National Chiao Tung University, Hsinchu 30010, Taiwan; E-Mails: cheyu.liu0801@gmail.com (C.-Y.L.); chienchunglin@faculty.nctu.edu.tw (C.-C.L.); hckuo@faculty.nctu.edu.tw (H.-C.K.)

**Keywords:** gallium nitride (GaN) microelectromechanical (MEMS) resonators, metal-organic chemical vapor deposition (MOCVD), epitaxial growth, piezoelectric

## Abstract

This work describes a novel architecture to realize high-performance gallium nitride (GaN) bulk acoustic wave (BAW) resonators. The method is based on the growth of a thick GaN layer on a metal electrode grid. The fabrication process starts with the growth of a thin GaN buffer layer on a Si (111) substrate. The GaN buffer layer is patterned and trenches are made and refilled with sputtered tungsten (W)/silicon dioxide (SiO_2_) forming passivated metal electrode grids. GaN is then regrown, nucleating from the exposed GaN seed layer and coalescing to form a thick GaN device layer. A metal electrode can be deposited and patterned on top of the GaN layer. This method enables vertical piezoelectric actuation of the GaN layer using its largest piezoelectric coefficient (*d*_33_) for thickness-mode resonance. Having a bottom electrode also results in a higher coupling coefficient, useful for the implementation of acoustic filters. Growth of GaN on Si enables releasing the device from the frontside using isotropic xenon difluoride (XeF_2_) etch and therefore eliminating the need for backside lithography and etching.

## 1. Introduction

Gallium nitride (GaN), typically grown on SiC, sapphire, or Si (111), is a piezoelectric material and is used as the transduction layer—sandwiched between a top and a bottom electrode—in bulk acoustic wave (BAW) resonators. However, unlike aluminum nitride (AlN), low-temperature sputtering of GaN on metals is not established, restricting its deposition or growth on specific substrates and making the fabrication of a metal-GaN-metal structure challenging. Because of such issues, different approaches have been taken to implement GaN-based piezoelectric transducers. The solutions that are sought so far include: (a) sputtering metal on the backside of the resonators, which requires release of the structure from the backside using deep reactive ion etching (DRIE) of the substrate [1,2,3,4]—DRIE is costly and usually not desired; (b) relying on lateral actuation without any bottom electrode—lateral excitation is not efficient as it relies on the weaker piezoelectric coefficient (d_31_) or the weaker lateral electric field, and yields lower electromechanical coupling; (c) using a two-dimensional electron gas (2DEG) as the bottom electrode [5,6,7,8,9] that is unique to III-V hetero-structures—the 2DEG is generally 20–30 nm below the surface of the structure due to restriction of lattice-mismatched epitaxial growth, considerably limiting the thickness of the active piezoelectric layer compared to the resonant stack and making it inefficient as the actuator. This work seeks a different solution using embedded bottom electrodes for piezoelectric actuation of GaN resonators. This technique enables frontside release of the resonant structure using xenon difluoride (XeF_2_), therefore eliminating the DRIE step.

**Figure 1:**
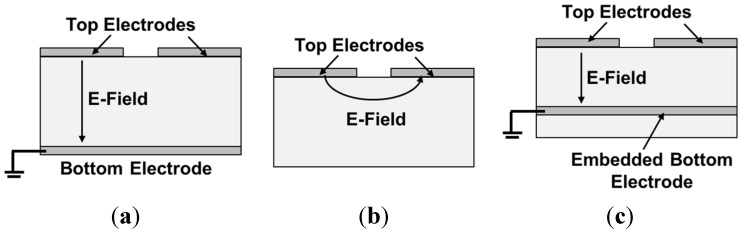
(**a**) Vertical electric field applied between the top and the bottom electrode, and (**b**) lateral electric field between two adjacent electrodes. (**c**) This work: electric field applied between the top electrode and an embedded meshed metal bottom electrode.

GaN thickness-mode resonators, most suitable for high-frequency applications, have been shown previously by our group at the University of Michigan. In [3] and [4], we demonstrated thickness-mode GaN BAW resonators with frequency × Quality factor (*f × Q*) values as high as 2.87 × 10^12^ and piezoelectric coupling coefficient (*K*_t_^2^) values of up to 1.7% [3]. The fabrication process was based on DRIE backside etching of the Si (111) handle layer followed by sputter deposition of the bottom electrode from the backside (Figure 1a). In [10], length-extensional resonance modes are excited based on lateral electric fields (Figure 1b). Even though high *Q*s are reported for the length-extensional resonance modes, the *K*_t_^2^ of such resonators is significantly lower due to the absence of a bottom electrode. More recently, a 2DEG layer induced at the AlGaN/GaN interface is used as the bottom electrode instead of a metal electrode. The mobility and sheet density of the 2DEG are typically about 1500 cm^2^/V·s and 10^13^ cm^−2^, respectively. Unlike metal electrodes, 2DEG has no mechanical loading effect on the resonator stack and can potentially result in higher mechanical *Q*s [11]. However, the location of 2DEG sheet is predetermined by the growth conditions and is commonly very close to the stack surface. For example, in [7] and [9], the 2DEG is only 20 nm below the resonator top surface. A 20 nm thick active piezoelectric layer is not an efficient actuator for excitation of a 1–3 μm thick GaN layer. In addition, the relatively low conductivity of 2DEG as compared to metals results in lower electrical *Q* for the resonator. Here, we use a third resonator architecture (Figure 1c) that has the advantage of having a highly conductive bottom metal electrode without requiring backside etching and electrode sputtering. The fabrication process is detailed in the next section.

## 2. Resonator Architecture

### 2.1. Regrowth of GaN on Embedded Metal Grids

In order to eliminate the need for backside processing of the wafer for releasing the resonant structure, resonant devices need to be released from the frontside. This requires a bottom electrode, embedded in the resonant structure during growth. However, as mentioned before, GaN cannot be directly grown on solid metal layers and the epitaxial growth has to start from a material with similar crystalline orientation. Using a meshed metal electrode on a GaN seed layer, the nucleation can still start from the bottom GaN layer, while a grid of metal is embedded in the resonant structure. The choice of metal is critical since it has to endure a high growth temperature (~1100 °C) and should not diffuse into the top GaN layers. For this purpose, tungsten (W) is used as the metal layer and is capped with a thin silicon dioxide (SiO_2_) layer to prevent its diffusion into the GaN device layer.

**Figure 2:**
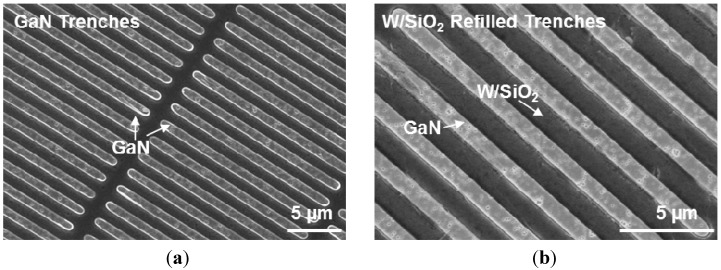
SEM images showing (**a**) 250-nm deep trenches made in GaN, forming a grid-like pattern; (**b**) trenches are refilled with W/SiO_2_. The darker regions are the W/SiO_2_ trenches, connected all through the sample, with GaN islands exposed to act as the seed layer for the GaN regrowth initiation. It is important for the GaN islands to have a fairly similar height as the W/SiO_2_ trenches to ensure smooth regrown GaN surface.

**Figure 3:**
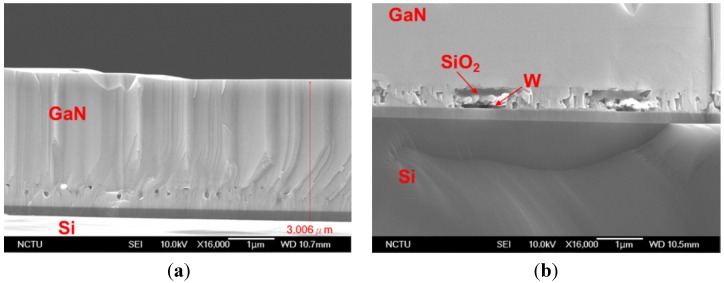
SEM Images of the cross-section of the regrown GaN, (**a**) on a reference GaN buffer layer of 500 nm thickness with no pattterns. The total thickness of the GaN stack is ~3 μm. (**b**) GaN regrown on W/SiO_2_ structures and GaN islands. The regrowth of GaN has well-coalesced and a uniform film is grown on top of the meshed metal electrode. W and SiO_2_ layers are marked.

The fabrication process starts with the growth of the buffer layer on a Si (111) substrate. In this work, we used a commercial 500 nm-thick unintentionally-doped (UID) GaN template grown on a Si (111) substrate purchased from Kyma Technologies [12]. Next, the GaN buffer layer was patterned and etched with 250 nm-deep trenches using a chlorine (Cl_2_) based plasma recipe (Figure 2a) and refilled with sputter-deposited W and evaporated SiO_2_ layers of ~120 nm and ~100 nm-thick, respectively (Figure 2b). The W/SiO_2_ patterning was done using a lift-off process. The W/SiO_2_ formed a pattern such that GaN can be partially exposed to serve as the seed layer for the subsequent GaN regrowth. The sample surface was cleaned of organic contaminants with oxygen (O_2_) plasma, acetone, and Isopropyl alcohol (IPA). Hydrogen peroxide (H_2_O_2_) wet surface cleaning was then performed to clean any trace of exposed W. Exposed W can diffuse into regrown GaN layer if it is not capped well with SiO_2_. The sample was returned to the metal-organic chemical vapor deposition (MOCVD) reactor to regrow the thick GaN layer. The regrowth was done at National Chiao Tung University (Taiwan), using a Veeco D75 MOCVD system. The growth temperature was 1070 °C, with a V/III ratio of 1500. Nitrogen (N_2_) and hydrogen (H_2_) were used as the environment gases with H_2_ as the carrier gas. The growth rate was ~2.8 μm/h. The thickness of the regrown GaN is measured to be ~2.5 μm (Figure 3).

### 2.2. Experimental Results

**Figure 4:**
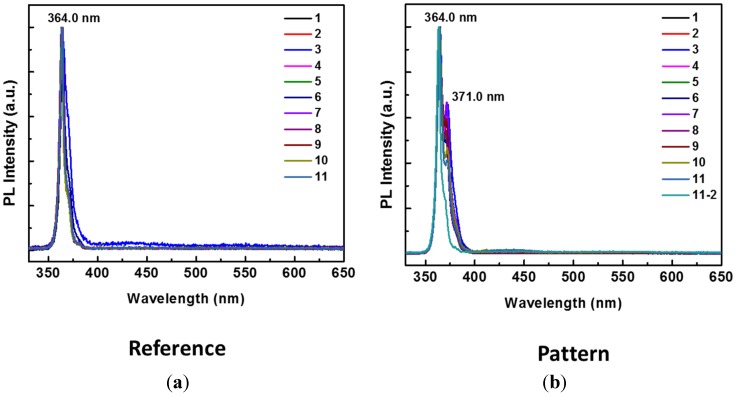
Room temperature photoluminescence (PL) measurement of 11 different points on (**a**) the reference sample, where GaN is grown on a thin GaN buffer layer without any patterns and embedded metals, or the reference, and (**b**) on the W/SiO_2_ patterned structures. The PL measurements clearly indicate the GaN peak (at 364 nm) is not degraded in (**b**).

The regrown GaN on the patterned seed layer is characterized and compared against GaN regrown on non-patterned GaN seed layer (reference). Photoluminescence (PL) measurements are taken to prove that the quality of GaN formed on the patterned structure is not degraded. Results shown in Figure 4 demonstrate that high-quality GaN layers can be grown on a patterned metal electrode. The ability to grow GaN on embedded metal electrodes opens up many exciting fields using Metal-GaN-Metal structures.

## 3. Resonator Fabrication Overview

**Figure 5:**
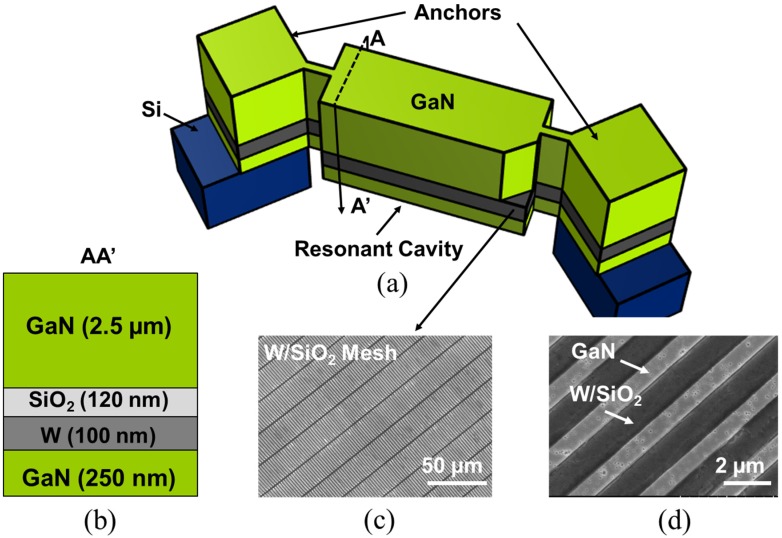
(**a**) An exemplary schematic of a GaN resonator with a thick GaN layer grown on W/SiO_2_ embedded electrodes. (**b**) The resonant stack schematic with a total thickness of ~3 μm. (**c**,**d**) SEM images of the GaN trenches before the GaN regrowth.

**Figure 6:**
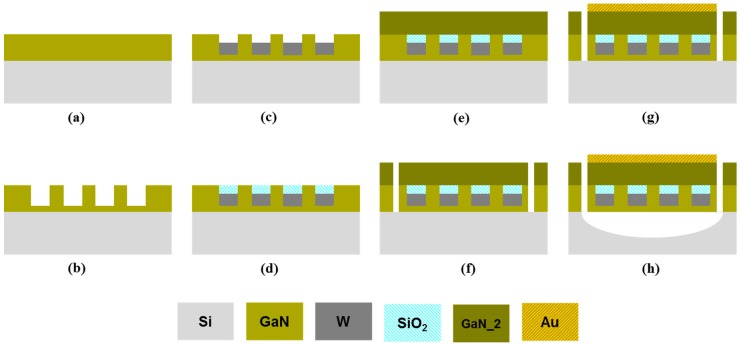
Fabrication steps of GaN resonator with embedded bottom electrode. (**a**) A thin layer of GaN is grown on a Si (111) substrate, (**b**) 250 nm deep trenches are made in the GaN layer using BCl_3_/Cl_2_ plasma etch, (**c**) the trenches are filled with sputtered W and (**d**) evaporated SiO_2_, (**e**) GaN device layer is regrown on the W/SiO_2_ mesh, starting from the bottom GaN seed layer, (**f**) trenches are made through the GaN layer to access the Si substrate, (**g**) the top metal is deposited on the device area, (**h**) the resonator is released with XeF_2_ isotropic etch.

As discussed in the earlier sections, one of the biggest challenges in the fabrication of GaN bulk acoustic resonators is the ability to epitaxially grow GaN on metal electrodes. Using the method shown in Section 2 and the stack shown in Figure 3, by depositing a top metal electrode on the regrown GaN and subsequently releasing the devices from the frontside using XeF_2_, GaN BAW resonators can be realized. An exemplary schematic of the proposed GaN resonator is shown in Figure 5a and the fabrication steps are discussed in detail in Figure 6. After realization of the stack shown in Figure 3, the thick GaN layer is patterned to form the contours of the resonator. Access to the Si substrate is realized through vias etched in GaN for the release step. The top electrode is then deposited on top of the GaN layer. The devices are finally released from the frontside using XeF_2_ that removes the Si substrate isotropically. Alternatively, cavity wafers (wafers with a cavity under the Si device layer) can be used instead of frontside isotropic release to fabricate free-standing devices.

## 4. Placement of the W/SiO_2_ Meshed Bottom Electrode

**Figure 7:**
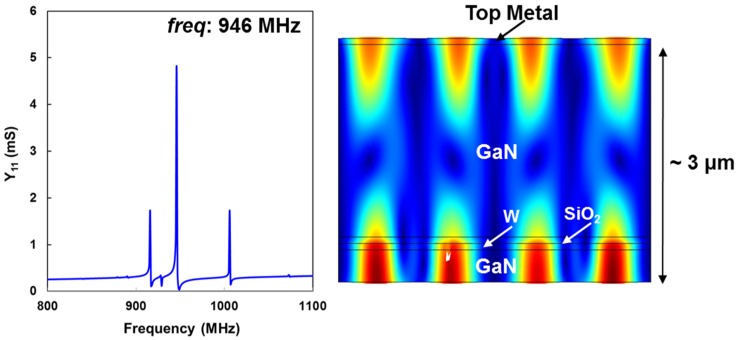
COMSOL [15] simulation of |Y_11_| response of a GaN bulk acoustic wave (BAW) resonator along with displacement profile of the thickness-mode resonance at 946 MHz.

One of the biggest advantages of our approach is the ability to optimize the bottom electrode location within the resonator stack. The placement of the bottom electrode affects several parameters in the resonator performance, such as *Q*, *K*_t_^2^, and temperature coefficient of frequency (TCF). Figure 7 shows the simulated admittance response of the resonator with the stack shown in Figure 5b. The admittance of the resonator is plotted around the thickness-mode resonance frequency along with the displacement profile of the same thickness-extensional mode.

**Figure 8:**
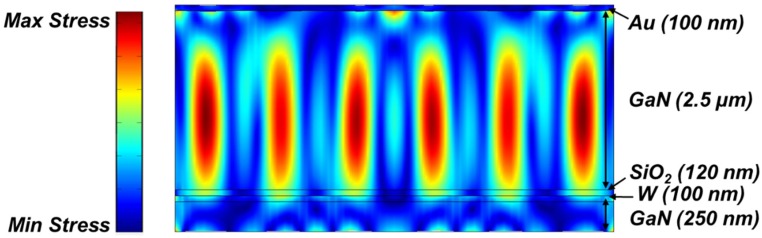
COMSOL simulation of the stress profile of the fundamental thickness-mode resonance at 946 MHz for the stack in Figure 5b. 120 nm thick embedded oxide layer is placed within the stack, fully compensating the temperature-induced frequency shifts of the fundamental thickness-mode resonance of the GaN piezoelectric resonator. Assuming a TCE value of -60 ppm/K for GaN, and TCE of +160 ppm/K for SiO_2_ [13], TCF of the resonator is simulated to be ~−5 ppm/K for the stack shown above.

Another advantage of using embedded electrode with the SiO_2_ diffusion barrier is that the SiO_2_ can also act as a passive temperature compensation layer. Passive compensation is realized by using materials with opposite temperature coefficient of elasticity (TCE). Most materials (e.g., Si, AlN, GaN) have a negative TCE, and therefore their resonance frequency decreases with an increase in temperature. SiO_2_, unlike GaN, has a positive TCE (TCE of SiO_2_: ~+160 ppm/K, TCE of GaN: ~−60 ppm/K) and can be used to cancel the temperature-induced frequency drift in GaN resonators [13]. Both the volume and location of SiO_2_ structures play a critical role in determining the TCF of a resonator. It is shown in [14] that placing embedded SiO_2_ structures at the location of maximum stress is most effective in reducing TCF values. In [13], we have shown GaN micromechanical resonators with a 400 nm thick blanket SiO_2_ on the top surface to reduce the value of TCF to ~−15 of ppm/K. Using the structure in this work, even with a thinner SiO_2_ layer, which is placed at high stress locations, significantly higher levels of temperature compensation can be achieved (Figure 8). More specifically, the first-order TCF of the GaN resonator with our proposed architecture can be canceled out for the fundamental thickness-mode resonance (Figure 8).

## 5. Conclusions

This work demonstrated a novel structure for GaN bulk acoustic resonators using vertical electric field for efficient piezoelectric actuation. In this approach, DRIE and backside metal sputtering is not used in the fabrication process as a bottom metal electrode is embedded in the GaN stack during the growth process and the GaN structural layer is released from the frontside with XeF_2_ etching of Si substrate. Using this approach, the placement of the bottom electrode can be optimized to maximize the charge pickup and displacement at resonance. Furthermore the embedded silicon dioxide protective layer used to prevent diffusion of tungsten in the regrown GaN layer can also be used for temperature compensation.
